# Impact of Liver and Pancreas Diseases on Nutritional Status

**DOI:** 10.3390/nu13051650

**Published:** 2021-05-13

**Authors:** Pablo Cañamares-Orbis, Vanesa Bernal-Monterde, Olivia Sierra-Gabarda, Diego Casas-Deza, Guillermo Garcia-Rayado, Luis Cortes, Alberto Lué

**Affiliations:** 1 Unidad de Gastroenterología, Hepatología y Nutrición, Hospital Universitario San Jorge, 22004 Huesca, Spain; pablocanamaresorbis@gmail.com; 2 Servicio de Aparato Digestivo, Hospital Universitario Miguel Servet, 50009 Zaragoza, Spain; vbernalm@gmail.com (V.B.-M.); osierra@alumni.unav.es (O.S.-G.); diegocasas8@gmail.com (D.C.-D.); 3 Instituto de Investigación Sanitaria (IIS) Aragón, 50009 Zaragoza, Spain; guillermogarcia7@hotmail.com (G.G.-R.); lucorga@gmail.com (L.C.); 4 Service of Digestive Diseases, Hospital Clínico Universitario Lozano Blesa, 50009 Zaragoza, Spain

**Keywords:** liver cirrhosis, malnutrition, malabsorption, vitamins, minerals, sarcopenia, liver transplant, pancreatic exocrine insufficiency, nutritional assessment

## Abstract

Liver and pancreatic diseases have significant consequences on nutritional status, with direct effects on clinical outcomes, survival, and quality of life. Maintaining and preserving an adequate nutritional status is crucial and should be one of the goals of patients with liver or pancreatic disease. Thus, the nutritional status of such patients should be systematically assessed at follow-up. Recently, great progress has been made in this direction, and the relevant pathophysiological mechanisms have been better established. While the spectrum of these diseases is wide, and the mechanisms of the onset of malnutrition are numerous and interrelated, clinical and nutritional manifestations are common. The main consequences include an impaired dietary intake, altered macro and micronutrient metabolism, energy metabolism disturbances, an increase in energy expenditure, nutrient malabsorption, sarcopenia, and osteopathy. In this review, we summarize the factors contributing to malnutrition, and the effects on nutritional status and clinical outcomes of liver and pancreatic diseases. We explain the current knowledge on how to assess malnutrition and the efficacy of nutritional interventions in these settings.

## 1. Introduction

Liver and pancreatic diseases have significant consequences on nutritional status. Malnutrition affects between 20% and 50% of patients with liver disease, and this rate could rise to 80% for patients with decompensated cirrhosis [1]. In chronic pancreatitis, up to 90% of patients have exocrine pancreatic insufficiency (EPI) twelve years after diagnosis, 40% of patients are underweight, and 17% have sarcopenia [2,3,4]. Frequently, the effects on nutritional status remain undiagnosed until the disease is already in an advanced stage, when it is challenging to manage.

In the last year, the impact of malnutrition on clinical outcomes has been well established. In patients with liver cirrhosis, malnutrition increases the incidence and severity of decompensations, influences the immune response, reduces muscle mass, deteriorates functional status and quality of life, and is associated with increased mortality [5,6,7]. In particular, malnutrition is related to the development and severity of hepatic encephalopathy [8] and has an impact on evolution after liver transplantation [9]. In patients with pancreatic disease, malnutrition is associated with increased mortality and hospitalization rates, a low quality of life, and poorer survival in patients with advanced pancreatic cancer [4,10,11,12].

Given these data, maintaining and preserving an adequate nutritional status is crucial and should be one of the goals of such patients. Recently, great progress has been made in this direction, and the relevant pathophysiological mechanisms have been better established. While the spectrum of these diseases is wide, and the mechanisms of the onset of malnutrition are numerous and interrelated, clinical and nutritional manifestations are common. Regardless of the etiology and the organ affected, the fundamental mechanisms of malnutrition in these diseases include poor dietary intake, malabsorption of micronutrients and macronutrients, and altered metabolism [13,14,15,16].

Even if the detrimental effects of malnutrition associated with liver and pancreatic diseases are well known, the scientific knowledge of a successful strategy to correct this manifestation is controversial, and the goal of contributing to such knowledge is difficult to achieve in the clinical practice [1,17].

In this review, we aim to describe the factors contributing to malnutrition and the effects of liver and pancreatic diseases on nutritional status and determine how to assess malnutrition. Further, we summarize the current knowledge on the management of malnutrition and the efficacy of nutritional interventions in these settings.

## 2. Consequences of Liver Disease on Nutritional Status

Liver fibrosis is the consequence of different inflammatory processes occurring in any chronic liver disease. Its progression determines the development of cirrhosis and portal hypertension. The natural history of cirrhosis is characterized by a compensated phase, with or without portal hypertension, and a decompensated phase characterized by the appearance of major complications, such as ascites, portal hypertensive bleeding, encephalopathy, and jaundice.

Malnutrition is frequent in patients with liver cirrhosis, which progresses in parallel with the worsening of the disease. Its etiology is multifactorial, given the great impact of liver disease on multiple processes related to nutrition [18]. In this section, we summarize the consequences of liver disease on nutritional status, focusing on the different components and functions that might be affected throughout the course of the disease (Table 1).

### 2.1. Impaired Dietary Intake

Anorexia is very common in patients with cirrhosis. It is caused by a mechanical effect, such as ascites, or by an imbalance between orexigenic and anorexigenic hormones (decrease of ghrelin and increase in leptin, respectively) [19]. Moreover, dietary restrictions imposed—sometimes quite rightly (sodium restriction for ascites) and sometimes based on false convictions (protein consumption restriction for encephalopathy)—may adversely limit dietary intake and cause taste alterations. The maintenance of an adequate nutritional status should prevail over dietary restrictions [20].

Regarding the etiology of liver disease, dietary intake is worse in alcoholic patients, given that alcohol represents their principal source of energy, rather than nutrient-rich foods. As a result, they develop nutrient deficiencies such as low serum levels of folate (B9), cobalamin (B12), and pyridoxine (B6), as well as macronutrient deficiencies.

### 2.2. Altered Macro and Micronutrients Metabolism

#### 2.2.1. Plasma Proteins

Hepatocytes play a central role in the production of plasma proteins, and the liver is the site where 80% of blood proteins are formed, except for gamma-globulin, which is produced by the reticuloendothelial system in the Kupffer cells. Plasma proteins include albumin (55%), globulins (38%), and fibrinogen (7%). They also include clotting factors, carrier and transport proteins, hormones, apolipoproteins, and other proteins involved in homeostasis.

Albumin is the principal plasma protein and modulator of the fluid distribution in compartments of the body, accounting for about 70–75% of the total plasma oncotic pressure. It also has other important roles, such as antioxidation, and immune-modulatory function, and the maintenance of homeostasis. A reduced serum concentration of albumin is a common feature in patients with cirrhosis. It has an adverse prognosis and occurs in parallel with the severity of liver cirrhosis, reaching a 60–80% reduction in advanced cirrhosis. Hypoalbuminemia results from both the decreased synthesis and complications due to the disease progression, such as ascites, which dilutes extracellular fluid protein content. Moreover, the sustained systemic inflammatory and pro-oxidant state induces structural changes of albumin that compromise the non-oncotic properties of the molecule [21].

#### 2.2.2. Vitamins and Minerals

The liver is a key storage site for several macro and micronutrients, including vitamin A, copper, manganese, iron, fatty acids, and glycogen, among others. It is also an important organ in terms of the production of binding, transport, and regulatory proteins required for micronutrient homeostasis and bile acids required for intestinal absorption. As a consequence, patients with chronic liver disease are at risk of the depletion of fat and water-soluble vitamins and minerals to a greater or lesser extent, depending on the etiology. For example, deficiencies of vitamin B12, folate, and zinc are the most well recognized symptoms of patients with alcoholic liver disease, whereas patients with cholestatic liver disease, deficiencies of fat-soluble vitamins prevail [14,15,16]. In end-stage liver disease, a lack of vitamins and minerals is nearly universal. Interestingly, vitamin D deficiency is present in 64% to 92%, regardless of the etiology of liver disease, and is associated with liver fibrosis and related to nonresponse to antiviral therapy for chronic hepatitis C [22]. In Table 2, the function of vitamins and minerals, their relationship with the liver, and the consequences of their deficit are summarized [23,24].

### 2.3. Energy Metabolism Disturbances

The human liver plays a central role in regulating fuel metabolism. The maintenance of glucose homeostasis, disposal of nitrogen by the urea cycle, and ketogenesis from fatty acids are some of the most important functions driven by the liver for maintaining internal homeostasis, and they are intimately linked. Liver disease leads to numerous metabolic disturbances. Firstly, due to the impairment of the ability to synthesize, store, and break down glycogen by hepatocytes, there is a decreased level of glycogenolysis and increased level of gluconeogenesis from muscle proteolysis, leading to a catabolism condition and sarcopenia. This not only leads to a decline in muscle mass, but also to a remarkable insulin resistance, which leads to glucose intolerance in up to 60% to 80% of patients, with cirrhosis and diabetes mellitus in 20% of cases. Moreover, it has been shown that the majority of energy is derived from fat oxidation. Conversely, in healthy subjects, fat oxidation accounts for only 40% of the total energy expenditure, after an overnight fast.

Secondly, there is a hypermetabolic state due to the increased production of cytokines that activates gluconeogenesis from muscle proteins and leads to a breakdown of muscle cells via autophagy and sarcopenia. Due to this, both patients with cirrhosis and acute liver diseases, such as acute hepatitis, have a high incidence of wasting and malnutrition. This hypermetabolic state is also responsible for hyperdynamic circulation and its consequences, such as bacterial translocation from the gut and the chronic inflammation state.

Finally, sedentariness also contributes to energy metabolism disturbances. Physical activity is an important determinant of muscle anabolism and the correct use of energy. In cirrhotic patients, sedentariness is a frequent characteristic consequence of many factors, such as ascites or other concomitant diseases, which perpetuate sarcopenia and its metabolic disorders, such as hyperammonemia and fat oxidation. This energy metabolism disturbance leads to an increase in energy expenditure, which has been reported to be associated with metabolic risk factors, including insulin resistance and high blood pressure, turning cirrhosis into a risk state of developing metabolic syndrome [25].

### 2.4. Increase in Energy Expenditure

In healthy people, the energy supply must balance the total energy expenditure (TEE) to maintain nutritional equilibrium, which includes a combination of resting energy expenditure (REE), physical activity expenditure, and food-related thermogenesis.

Specifically, TEE is composed of the energy costs of the processes essential for life (basal metabolic rate (BMR), 60–80% of TEE), of the energy expended in order to digest, absorb, and convert food (diet-induced thermogenesis, ~10%), and the energy expended during physical activities (activity energy expenditure, ~15–30%) [26].

REE represents the amount of energy expended by a person at rest. In practice, REE and BMR differ by less than 10%, so the terms can be used interchangeably. As mentioned above, it contributes to 60–80% of TEE, being the largest component of total daily energy expenditure both in healthy and pathological subjects [26].

An increased REE has been proposed to be of pathophysiological importance in liver disease, given that cirrhosis is a state of accelerated starvation. It has been described to be raised to 120% of the expected value in more than 15–30% of patients with liver cirrhosis, and the principal mechanisms responsible for this state include hypermetabolism, defined as measured REE > 20% above predicted RE, malnutrition, and immunosuppression [13,27].

### 2.5. Nutrient Malabsorption

Several factors can contribute to the malabsorption of nutrients in cirrhotic patients. One of them is portosystemic-shunting, which makes nutrients bypass the liver without metabolic processing. Another factor to consider is a drop in bile production due to impaired liver function. As a result, micelles formation is defective, and the absorption of long chain fatty acids through the usual lymphatic route is missing. This has pathophysiologic implications and can result in an excess hepatic storage of fat, which can reduce liver function and the systemic availability of fat for organic functions. Small intestinal bacterial overgrowth (SIBO) is very common in cirrhotic patients and may contribute to malabsorption. Colonic bacteria colonize the small bowel and impair microvilli function, digestive enzyme production, and intestinal barrier dysfunction, causing a disturbed absorption and metabolism of nutrients and affecting intestinal motility. SIBO may also be involved in bacterial translocation and infectious complications, such as spontaneous bacterial peritonitis [28]. Finally, drug-related malabsorption due to diarrhea (e.g., lactulose, antibiotics, or diuretics) or interference with fat absorption (e.g., cholestyramine) can also contribute to the malabsorption of nutrients. Moreover, decompensations and several complications of end stage liver disease, like over hepatic encephalopathy or spontaneous bacterial peritonitis (SBP), are directly or indirectly linked with the gut microbiota. Several studies have evaluated how microbiota changes in cirrhosis. Overall, widespread dysbiosis is observed in cirrhotic patients, with reduction in autochthonous taxa and increase in pathogenic ones. Particularly, reduced Bacteroidetes with increased Proteobacteria at the phylum level, increased Veillonella and Streptococcus spp., a significantly higher abundance of Enterobacteriaceae, but lower Lachonospiraceae, Ruminococcaceae, and Blautia (7a-dehydroxylating bacteria) in the cirrhosis group compared to controls [29].

### 2.6. Sarcopenia and Muscle Function

Sarcopenia is defined by a loss of muscle mass and decreased functional capacity. This complication may be present in the early stages of liver disease, but it is more frequent and severe in the end-stage disease, with a prevalence of nearly 60%. It is associated with a higher mortality, increased hospital admissions, worse post-liver transplant outcomes, decreased quality of life, and increased risk of other complications associated with cirrhosis [30].

Again, multiple factors are thought to be involved in the development of sarcopenia, some of which are common to other components of malnutrition previously mentioned, such as an altered carbohydrate and lipid metabolism, malabsorption, hypermetabolism, and anorexia. However, the most well-documented factor that contributes to sarcopenia is hyperammonemia. Hyperammonemia is a metabolic condition characterized by raised levels of ammonia, a nitrogen-containing compound, that is a potent neurotoxin. Ammonia levels rise if the liver is unable to metabolize this toxic compound as a result of an enzymatic defect or hepatocellular damage. Normal levels of ammonia vary according to age. In healthy adults the normal level is less than 30 micromol/L [31]. The decrease in the hepatic clearance of ammonia is compensated by the muscle clearance, whereby energy and proteins are expended, and hence, muscle breakdown is increased. It also induces the up-regulation of myostatin, the main muscle growth inhibitory factor, which, together with the descent of IGF-1 and testosterone, the main muscle growth-promoting factors in liver cirrhosis, leads to sarcopenia [32].

Apart from the loss of muscle mass, patients with liver cirrhosis have a decreased muscle function due to a mitochondrial dysfunction and direct modifications of contractile proteins. Additionally, the increased muscle breakdown and reduced muscle quality, as determined by the fat infiltration, observed through imaging, contributes to the poor physical condition.

### 2.7. Metabolic Osteopathy

Osteoporosis and osteopenia are common complications in patients with cirrhosis, with a prevalence of approximately 12–55% higher than in healthy people [33]. First is a systemic bone disease, which is characterized by a low bone mineral density (BMD), micro architectural malformation, and susceptibility to fracture. Osteopenia is a low-grade osteoporosis.

In chronic liver diseases, bone loss refers to a decrease in bone formation and increase in bone resorption. The main mechanisms underlying osteoporosis in patients with chronic liver disease are vitamin K deficiency, vitamin D and calcium metabolism alterations, hormonal dysregulation, and proinflammatory cytokines related to “leaky gut syndrome” and IGF-1 deficiency [34]. These abnormalities differ depending on the etiology: in cholestatic liver disease, deficiencies of vitamin K and D represent the main cause of metabolic osteopathy; in hemochromatosis, there is an associated hypogonadism that can explain this condition; in Metabolic Associated Fatty Liver Disease (MAFLD), viral hepatitis and alcoholic liver disease, the increase in proinflammatory cytokine production represents the pathophysiological mechanism.

### 2.8. Interplay between MAFLD and Diet

The prevalence of MAFLD is increasing as the rate of obesity rises as well as sedentary lifestyles and other components of metabolic syndrome. Fortunately, only a small percentage of patients develop inflammation and subsequently fibrosis and chronic liver disease. Obesity does not rule out malnutrition. In fact, several studies have described a significant association between sarcopenia and MAFLD, independent of obesity and insulin resistance [35]. Moreover, MAFLD is also present in 7% of normal-weight (lean) persons.

The underlying pathophysiological mechanism of MAFLD remains unclear. The “multiple hit” hypothesis considers multiple insults acting together on genetically predisposed subjects, such as insulin resistance, hormones secreted from the adipose tissue, nutritional factors, gut microbiota and genetic and epigenetic factors [36]. Recently, a complex interplay between the gut microbiota, intestinal barrier and nutrition has been described. The dietary factors may alter the gut microbiota and intestinal barrier function, directly affecting hepatic organelles and cell-to-cell communications, favoring the occurrence of metabolic endotoxemia and low-grade inflammation and generating an adverse microenvironment which could in which several hepatocytes select anti-apoptotic programs and mutations that may allow survival and proliferation [37,38]. These facts may facilitate MAFLD progression from simple steatosis to nonalcoholic steato-hepatitis (NASH) and cirrhosis and development of hepatocellular carcinoma (HCC) [37,38].

## 3. Nutritional Assessment of Patients with Chronic Liver Disease

### 3.1. Nutritional Screening and Risk of Malnutrition

Nutritional screening should be performed in all patients with liver cirrhosis, especially if they have portal hypertension or liver failure.

The European Society for Clinical Nutrition and Metabolism (ESPEN) states that screening tools should be simple and quick, and untrained personnel should be able to administer them. A good screening tool has to be easy to apply and have a reasonable specificity, but above all, it should have an excellent sensitivity [39].

There are a large number of nutritional screening tools, each with its own strengths and weaknesses. Table 3 shows the most frequently used tools [40,41,42,43,44,45,46]. There are variables common to most of them, such as BMI and weight loss, and other ones which vary, such as muscle mass assessment, food intake, appetite, etc. In recent years, specific tools have been developed for patients with liver diseases, such as the Royal Free Hospital Nutritional Prioritizing Tool (RFH-NPT) and Liver Disease Undernutrition Screening Tool (LDUST). All general tools that take into account BMI or weight loss as a variable may be inaccurate due to the presence of oedema and/or ascites, which is very prevalent in the liver cirrhosis patient population. What LDUST and RFH-NPT have in common is that they seek to exclude or limit the impact of weight gain through fluid retention, as shown through anthropometric assessment. RFH-NPT has shown a higher diagnostic and complication predictive capacity. In addition, LDUST has a higher degree of subjectivity, since at least two of the questions depend on the patient’s assessment and may have a high degree of variability.

As for the other tests, several studies have recently been published showing that the Mini Nutritional Assessment Short Form (MNA^®^-SF) may have an important role in patients with liver cirrhosis. The limited mobility of these patients and their neuropsychiatric problems may make this test a useful tool.

Finally, in patients on surgery waiting lists, the CONtrolling NUTritional status (CONUT) tool has an important capacity to predict complications and post-surgical mortality. In addition, it is quick and easy and may sometimes be automatically available for blood analyses.

### 3.2. Diagnosis of Malnutrition

**Figure 1:**
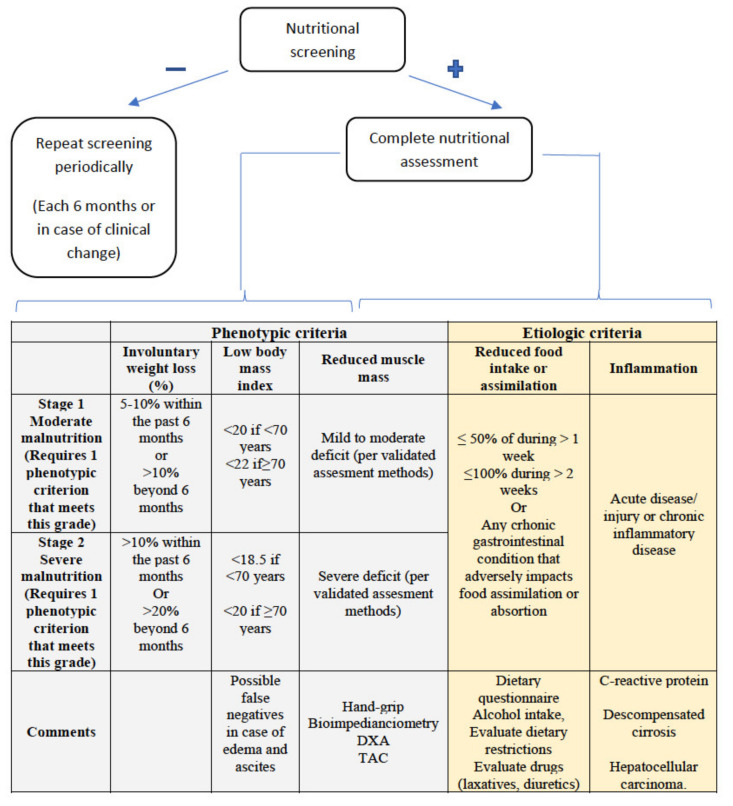
GLIM criteria for malnutrition diagnosis. At least one phenotypic criterion and one etiologic criterion are required.

When nutritional screening with any of the tools is positive, a comprehensive nutritional assessment should be carried out, which, in addition to confirming the diagnosis of malnutrition, allows for an assessment of the cause, severity, repercussions, deficits, and potential interventions to be carried out (Figure 1).

New diagnostic criteria for malnutrition have recently been published by the Global Leadership Initiative on Malnutrition, called the GLIM criteria [47], with the participation of the main nutrition societies worldwide. As can be seen in Figure 1, they consist of two groups of criteria, phenotypic and etiological, with the presence of at least one criterion from each group being necessary to establish a diagnosis of malnutrition. Severity (moderate or severe) is established according to the phenotypic criteria. According to the GLIM criteria, malnutrition affected 38.1% of patients with cirrhosis, being severe in 22% of the patients in a recently published study [48]. Using this tool as a guide, we will now discuss some particularities of the assessment of these points in patients with cirrhosis of the liver.

#### 3.2.1. Assessment of Reduced Intake

There are several ways to assess dietary intake, some more sensitive than others. The simplest way is to ask the patient directly about what the proportion of their usual intake they are currently eating. Previously, the existing criteria established different cut-off points and also took into account the time of evolution. The suggestion of the GLIM group is to set at least 50% of energy requirements for at least one week or any reduction for at least 2 weeks for any chronic GI condition that adversely impacts food assimilation or absorption.

However, it is recommended to keep at least a 24-h nutritional diary, which allows for a more accurate and reliable assessment, or to apply one of the validated questionnaires, such as the “Patient Generated Subjective Global Assessment” [49].

In patients with liver cirrhosis, it is particularly important to stress the importance of protein-rich food consumption and to assess the impact of alcohol consumption on the quantity and quality of a patient’s diet.

#### 3.2.2. Weight Loss and Body Mass Index

In almost all screening tools and in virtually all comprehensive assessment methods or diagnostic criteria, weight loss and BMI play an important role. However, their assessment is particularly difficult in patients with liver cirrhosis, since, as mentioned above, they can be inaccurate due to the presence of fluid, either in the form of ascites or edema.

Some previous diagnostic criteria, such as the ESPEN criteria [50], attached great importance to BMI, making them insensitive in patients with a high baseline BMI or who might have a falsely elevated BMI, like patients with cirrhosis or heart failure. The GLIM criteria, on the other hand, do not require this item to be met, allowing a diagnosis of malnutrition to be made in this group of patients, even though they may have a normal BMI or no current weight loss.

#### 3.2.3. Muscle Mass and Body Composition

Among the complications associated with malnutrition in patients with liver cirrhosis, sarcopenia is particularly relevant. However, the assessment of body composition in patients with fluid overload can be particularly complex. There are several described methods that can be used in cirrhotic patients, such as bioimpedance [51], or dual-energy X-ray absorptiometry (DXA) [52]. An indirect, but more widely available, method is the assessment of muscle strength by hand-grip, although its correlation with the stage of cirrhosis is unclear [53]. In terms of cut-off points, there are different recommendations according to the different societies, which should be adjusted for age and sex.

As a complement to these techniques, which can be influenced by water retention, computed axial tomography allows for a more objective assessment of muscle mass, although with the disadvantage of the associated irradiation [54]. Moreover, the cut-off points are not properly established.

#### 3.2.4. Disease Burden/Inflammation

The presence of an inflammatory condition is the most difficult criterion to assess in patients with chronic disease. According to the GLIM criteria consensus document, the mere presence of a chronic disease, such as a neoplasm or liver cirrhosis, is not a sufficient condition to meet this criterion, and it is necessary to demonstrate the progression or decompensation of the disease, for which clinical, radiological, or analytical elements, such as C-reactive protein, can be used. The presence of an acute condition, such as an infection or another similar factor, would meet this criterion.

Within the nutritional assessment of patients with cirrhosis of the liver, certain particularities must be taken into account with respect to the general population. While macronutrient deficiencies have to be assessed as in other patients, patients with cirrhosis are at increased risk of micronutrient deficiencies, such as zinc and magnesium due to diuretic use, as well as vitamin A or vitamin D. The particular characteristics of patients with alcohol consumption, which is very prevalent in patients with cirrhosis of the liver, must also be taken into account.

In summary, the screening and diagnosis of malnutrition in patients with liver disease is complex and influenced by factors intrinsic to the cirrhosis itself. Alterations in body composition mean that global assessment must be adapted in order to be reliable. Therefore, the development of specific tools may be an important advance in nutritional assessment. As for the new GLIM criteria, their validity and prognostic ability remains to be demonstrated, although their lower dependence on BMI make them potentially interesting.

## 4. Nutritional Intervention in Liver Disease

In patients with liver cirrhosis, nutritional intervention aims to supply at least 35 kcal/Kg/day (in non-obese patients) and a protein intake of 1.2–1.5 g/Kg/day, or even more than 1.5 g/Kg/day if sarcopenia is already present [1,55]. The main nutrition strategies to achieve these goals include nutritional counselling, frequent feeding, and nutritional supplementation.

In a retrospective study that included 232 patients with liver cirrhosis, patients that received nutritional counselling through teaching sessions given by a multidisciplinary team (including physicians, nurses, pharmacists, and dieticians) showed improved survival rates and an improved quality of life [56]. Therefore, nutritional counselling is strongly recommended for patients with chronic liver diseases [57]. On the other hand, in patients with liver cirrhosis, the daily intake should be split into six meals or snacks, and the late-evening snack is essential. The late-evening snack shortens nocturnal fasting and decreases skeletal muscle proteolysis, thus improving quality of life [58]. In a meta-analysis that included eight studies and 341 patients with cirrhosis, a late-evening snack improved liver biochemical parameters and liver dysfunction [59].

Nutritional supplementation includes oral nutritional supplements—mainly branched chain amino acid (BCAA) supplements, enteral nutrition (EN), and parenteral nutrition (PN). Three randomized clinical trials (RCTs) [60,61,62] with BCAA showed beneficial results in cirrhotic patients. In one RCT with 174 patients with advanced cirrhosis, one-year supplementation with BCAA prevented progressive hepatic failure and decreased the hospital admission rate [60]. In another RCT, the administration of 12 g/day of BCAA for two years improved the event-free survival, serum albumin concentration, and quality of life of patients with decompensated cirrhosis [61]. Finally, a third multicenter RCT, developed in Spain, showed that a supplement of 30 g of BCAA showed an improvement in neuropsychological tests and an increase of muscle mass in patients with cirrhosis and a previous episode of hepatic encephalopathy (HE) [62]. Consistent with these results, the ESPEN guidelines on clinical nutrition in liver diseases recommend long-term treatment with oral BCAA in patients with advanced cirrhosis in order to improve their clinical evolution [57].

EN has been proposed in malnourished cirrhotic patients admitted to hospital, but systematic meta-analyses have not shown relevant positive results in terms of survival [63,64], so the routine use of EN in hospitalized cirrhotic patients is not supported. However, in malnourished cirrhotic patients who are unable to obtain correct dietary intake (even with oral supplements), a short treatment with EN should be performed [1,55,57]. In these patients, a nasogastric tube can be placed, even in the presence of esophageal varices. PN is recommended in cirrhotic patients who cannot receive adequate oral and/or EN, for example, in patients with intestinal ileus [57]. The composition of a PN solution in cirrhotic patients can be the same as that of a standard solution, because specific solutions, like the BCAA-enriched solution, did not show better results in terms of survival or other outcomes, such as quality of life or nutritional parameters [65].

In addition to nutritional supplementation, exercise is an important factor in preventing sarcopenia in cirrhotic patients [1]. Despite the absence of large studies, some clinical trials published in recent years showed hopeful conclusions. In a small prospective study, eight weeks of supervised exercise improved the aerobic capacity and muscle mass and decreased fatigue of patients with Child–Pugh class A or B cirrhosis [66]. Besides, in another study, moderate exercise for 16 weeks reduced the body weight and the hepatic venous pressure gradient in overweight/obese patients with cirrhosis [67]. As a general recommendation, exercise should include aerobic and resistance actions and should have a mild duration, for example, about 30–60 min [55,68].

Other conditions requiring particular management include obesity in cirrhotic patients, HE, and acute alcoholic hepatitis. In obese patients with cirrhosis, a reduction >5–10% of body weight is associated with a decrease of the disease progression [69]. In these obese patients, a strategy of exercise and a hypocaloric diet (between 500 and 800 Kcal/day) is recommended to obtain this reduction of body weight. Compliance with a calorie-restricted diet over the long term is associated with the mobilization of liver fat and an improvement in cardiovascular risk. The specific macronutrient composition of the diet appears to be less relevant than the sustained weight loss. However, diet should incorporate an adequate amount of protein (>1.5 g/kg/day) to avoid a loss of muscle mass [1].

Regarding HE, in the past, some studies with methodological flaws suggested that decreasing protein intake in patients with HE showed better outcomes. Nevertheless, more recent and better studies have not confirmed these results, and protein restriction is now considered to be detrimental both in patients with acute HE and those with chronic HE [1,57]. In general, vegetable and dairy protein is better tolerated than meat protein and can develop a prebiotic and laxative action [1]. BCAA supplements have been documented to promote muscle protein synthesis and improve muscle mass loss. Both of these effects are involved in the pathophysiology of HE, thus establishing the rational basis for its use in HE. In addition, in patients with HE, BCAA supplements are recommended, because they have a beneficial effect on overt HE, according to a Cochrane meta-analysis including 16 RCTs. Unfortunately, BCAA supplements had no effect on mortality [65].

Moreover, recent data published by Ericksen R.E. et al. suggest a positive correlation between BCAA intake and cancer risk in humans [70]. In this study, transcriptomic and metabolomic analysis of primary HCCs and animal liver cancer models also identified an important role for BCAA catabolism in tumor development, progression, and growth [70]. Specifically, the loss of BCAA catabolism and accumulation during carcinogenesis lead to a stimulation of mTORC1 activity, which promotes HCC development and progression in mice models [70]. Furthermore, the authors observed that BCAA catabolic enzyme expression predicts tumor aggressiveness and patient survival and that dietary BCAAs correlate with tumor burden in mice and cancer mortality in humans [70]. Other results also suggest that BCAAs may mediate pathways related to cancer development and progression, possibly due to their involvement in insulin metabolism [71]. Nowadays, this safety concern could represent a limitation for BCAA supplementation therapy.

Severe acute alcoholic hepatitis is a life-threatening condition with a high mortality, and EN could play a relevant role. In an RCT, 71 patients with severe alcoholic hepatitis were randomized to receive prednisolone or enteral tube feeding for 28 days. The mortality during treatment was equal in both groups, but occurred earlier with EN. However, patients in the prednisolone group had a higher rate of infections, which was associated with a higher mortality during a one-year follow-up [72]. In another study, 136 patients with alcoholic hepatitis were randomized to receive EN plus methylprednisolone or conventional nutrition plus methylprednisolone. No significant difference between the treatments in terms of 6-month mortality was shown, although there may be confounding factors, like active alcohol intake. Nonetheless, patients in the study with a daily calorie intake of less than 21.5 kcal/kg/day had a lower survival [73].

Additionally, in patients with chronic liver disease, micronutrient and vitamin deficiencies are frequent and are associated with hepatic dysfunction. Therefore, confirmed or clinically suspected deficiencies of micronutrients or vitamins must be treated [1,57]. Specifically, a deficiency of vitamin D is very common in cirrhotic patients and should be evaluated in all these patients [74]. If vitamin D levels are below 20 ng/mL, this deficiency should be corrected to achieve vitamin D levels above 30 ng/mL [1]. Other vitamin deficiencies, such as vitamin K in cholestatic diseases or vitamin B in alcoholic cirrhosis, should also be considered and treated. In addition, zinc deficiency is associated with HE, changes in taste and smell, and hair loss [55,75]. Zinc supplementation could cause an improvement in the taste and palatability of food, but the data on mental activity are not conclusive [76].

Finally, other nutritional interventions for sarcopenia, frailty, and malnutrition are emerging [55]. A recent 1-year clinical controlled trial of intramuscular testosterone in male patients with cirrhosis and low serum testosterone demonstrated that testosterone safely increases muscle mass without a clear effect on muscle function [77]. Larger-scale investigations are warranted, before this is implemented into routine clinical practice.

Reducing portal pressure by transjugular intrahepatic portosystemic shunt (TIPS) placement may have beneficial effects on muscle mass and decrease mortality in patients, showing an improvement of sarcopenia after the TIPS placement [78]. Nevertheless, caution should be exercised when performing TIPS in malnourished patients with cirrhosis, as sarcopenia is a risk factor for the development of HE and acute-on-chronic liver failure after TIPS placement.

## 5. Impact of Malnutrition and Its Therapy on Liver Transplantation

Liver transplantation (LT) has substantially changed the prognosis of chronic liver disease, with liver cirrhosis being the most common indication of LT worldwide. The amelioration of the immunosuppressive regimens and surgical techniques has progressively improved the outcome of these patients, and the survival rate after LT is nowadays 70–80% at five years [9]. Most LT centers use the model of end-stage liver disease (MELD) to prioritize LT according to the MELD score (based on serum bilirubin, creatinine, and INR), which favors the transplantation of the sickest patients and has reduced the mortality of patients on the waiting list. At the same time, it has been clearly shown that there is no benefit from transplantation when transplant patients have MELD scores lower than 15 [79]. However, there are certain limitations of this score, among which are a lack of incorporation of an assessment of the nutritional status of patients. For this reason, several authors have proposed the inclusion of nutritional parameters in the MELD score, such as sarcopenia, in order to improve the prediction of mortality in patients with cirrhosis. The importance of sarcopenia was reflected by the fact that the presence of sarcopenia is equivalent to adding 10 points to the MELD score [80,81].

Alterations in nutritional status are frequent in patients with advanced liver diseases, and the prevalence is considerably higher in patients with a more severe liver impairment [82]. In fact, the high rate of malnutrition in these patients contributes to the prevalence of sarcopenia or frailty being greater than 50% [83].

Malnutrition among patients awaiting liver transplantation is multifactorial. Situations like fasting are often required before propaedeutic tests and invasive procedures, such as paracentesis and esophageal varices ligation. A poor dietary intake is a common consequence of restrictive diets, like low-sodium diets in ascites, or an inadequate indication of a restrictive protein diet for preventing liver encephalopathy events. Early satiety and abdominal pain can be associated with large ascites. Furthermore, it is possible that there is an association of maldigestion and malabsorption with the gastrointestinal tract or pancreatic disease, as well as the side-effects of drug therapy, like lactulose, which can produce abdominal bloating or diarrhea [84,85].

The impact of malnutrition on increased morbidity and mortality has been reported by different studies, showing a negative impact after LT, so the presence of malnutrition is an indicator of an unfavorable outcome after LT [86]. Recipients’ malnutrition was found to be associated with an increased operative blood loss and infections, length of stay in the intensive care unit (ICU), and total hospital charges after LT [80,81,87,88]. A recent meta-analysis evaluating the impact of CT-assessed sarcopenia, including 3803 patients, showed an independent association between sarcopenia and wait-list and post-LT mortality, without employing the MELD score [89]. Despite this evidence, it is generally agreed that LT should not be denied, even in highly malnourished cirrhotic patients [90].

On the other hand, the prevalence of obesity and metabolic syndrome is increasing among LT candidates, particularly in metabolic-associated fatty liver disease, which has become an increasing indication of LT. Severe obesity (body mass index ≥ 40 kg/m^2^) prior to liver transplantation is associated with an increased mortality due to infectious complications, cardiovascular disease, and cancer, and it could be a relative contraindication to LT [91,92]. While less apparent, malnutrition and muscle wasting may be present in cirrhotic obese patients, and this condition is identified as “sarcopenic obesity”. The reduction in skeletal muscle mass has been suggested to be an independent predictor of survival, quality of life, outcome, and response to stress and surgery [93]. Furthermore, several metabolic complications related to weight gain and immunosuppression are developed in long-term post-LT. The risk of arterial hypertension, dyslipidemia, and diabetes mellitus incidence increase after surgery, along with impact outcomes and survival [94].

Whether the presence of a compromised nutritional status plays a role as an independent risk factor in the outcome of LT, screening for malnutrition and sarcopenia is recommended in cirrhotic patients evaluated for LT [1,9,92]. Unfortunately, malnutrition is frequently overlooked in LT because nutritional evaluation is not routinely carried out in clinical practice, which highlights the need to incorporate nutrition specialists into a multidisciplinary LT team [95].

In malnourished cirrhotic patients, the risk of postoperative morbidity and mortality is increased after abdominal surgery [93]. The pre- and postoperative recommendations to avoid malnutrition are similar to those given in cirrhotic patients, who do not expect LT, with a total energy intake of 30–35 kcal/kg/day and a protein intake of 1.2–1.5 g/kg/day. However, obese patients can be given a lower total energy intake of 25 kcal/kg/day and an increased protein intake of 2–2.5 g/kg/day [1,57].

Despite the importance of nutritional therapy for patients waiting for LT, no clear data are therefore available to support the efficacy of oral nutritional supplementation (ONS) in improving the clinical outcomes in these patients, so standard nutrition regimens shall be used [57]. Nutritional counselling plus ONS and nutritional counselling alone were equally effective in cirrhosis patients awaiting transplantation [96]. A combined meta-analysis of different interventions, like glutamine, BCAA supplements, and post-LT parenteral nutrition containing fish oil-derived long-chain n-3 PUFAs or ω-3 fatty acids, reported overall beneficial effects in terms of morbidity, decreased infections, length of hospital stay, and improved liver function, but no significant difference in survival was observed [97,98]. Other interventions in LT to ameliorate nutritional support, like vitamin D supplementation [99] or physical activity, for improving the muscle mass in LT candidates are proposed [100].

In deceased donor transplantation, it is not possible to predict when a patient will receive a LT, so aggressive early post-operative nutrition support (by the enteral route if possible) should be allocated to patients who are undernourished, especially when it is anticipated that patients will be unable to eat for more than two days or patients cannot maintain an oral intake above 60% of the recommended intake for more than 10 days [93]. Furthermore, after LT, normal food and/or enteral nutrition should be initiated within 12–24 h postoperatively to reduce the infection rate [57].

Additionally, in liver surgery, the adoption of enhanced recovery after surgery (ERAS) protocols improves morbidity and reduces the length of hospital stay, when, among other measures, patients are given carbohydrates containing clear liquids up to 2 h preoperatively and are fed early and mobilized [1,57,101].

## 6. Normal Pancreatic Physiology and Mechanisms of Malnutrition

The pancreas is a gland located in the upper hemiabdomen and is about 12 to 20 cm in size. It is divided anatomically into the head, the uncinate process, the neck, the body, and the tail. The head is closely related to the duodenum, which facilitates the exocrine function of assisting digestion through a duct and glandular system, which makes up 85% of the gland. In addition, the pancreas has another endocrine function of controlling the secretion of various hormones and regulating blood glucose, which represents only 2% of the pancreas.

The pancreatic exocrine functional unit is the acinus and the ductal system. Secretion is stimulated by multiple hormones and neurotransmitters, including secretin, vasoactive intestinal peptide (VIP), and acetylcholine (Ach). The main inorganic components of pancreatic juice are water, sodium, chlorine, and bicarbonate. The secreted pancreatic enzymes are amylases, lipases, and proteases, which are secreted inactively into the ductal system to be activated in the duodenal lumen by an enterokinase that is formed in the intestinal mucosa. In summary, the function of amylase is the digestion of carbohydrates (starch, glycogen, etc.), the lipase function is to facilitate the digestion of fats (neutral fats, cholesterol esters, etc.), and proteases, such as trypsin or chymotrypsin, play a role in the degradation of proteins into smaller amino acids.

The pancreatic digestive function is a complex process that involves the initiation of ingestion, stimulation through neurotransmitters, hormonal secretion, and the motility of gastric emptying.

It is divided into two phases: inter-digestive secretion and digestive secretion. The inter-digestive secretion promotes the cleansing of the excretion system, while the digestive secretion is divided into 3 phases: cephalic, gastric, and intestinal. The cephalic phase occurs when ingestion begins, even before the food reaches the stomach, which causes nerve stimulations to release Ach, thus stimulating the synthesis of pancreatic enzymes inside the acini. The gastric phase is stimulated by gastric distention, and the formation and secretion of small amounts of pancreatic enzymes continues into the acini and pancreatic ducts. The third and most important phase is the intestinal phase, which begins when the acid chyme formed in the gastric body, which can reach a very low pH (as low as 1 or 2), enters the duodenal lumen in a progressive manner, controlled by the gastric emptying. At this time, the pancreatic secretion is triggered by the action of the hormone, secretin (polypeptide formed by the S cells of the duodenum and jejunum), which causes a massive secretion of bicarbonate and water from the ductal system into the intestinal lumen, thereby causing an increase in the pH to 7 or 8. Pancreatic enzymes are inactivated when the pH falls below 3 [102], so this rise in pH is important. Likewise, another hormone, cholecystokinin (a polypeptide formed by the I cell of the duodenum and jejunum), causes the secretion of pancreatic enzymes and movements for the emptying of the gallbladder, thus progressively and homogeneously joining with the chyme from the stomach. Normal pancreatic physiologic functions are summed up in Table 4.

EPI is the mechanism that causes malnutrition in patients with pancreatic disease. EPI in adults occurs mainly in cases of chronic pancreatitis (CP), either after surgical resections of the pancreatic gland or as a complication after severe acute pancreatitis due to the absence of viable functional glandular tissue. Other diseases also develop EPI, such as cystic fibrosis (more frequently diagnosed in childhood), advanced celiac disease, diabetes mellitus, Crohn’s disease, or Zollinger–Ellison syndrome [2], through different mechanisms:

1. Decreased production and secretion of pancreatic lipase due to a reduction of the pancreatic parenchyma, which occurs in chronic pancreatitis, autoimmune pancreatitis, and in pancreatic resections.
2. Inaction of pancreatic lipase, which is usually due to the inactivation of lipase in excessively acidic environments, such as cystic fibrosis, where bicarbonate secretion is significantly reduced by the mutation in the cystic fibrosis transmembrane conductance regulator (CFTR) gene, so the intestinal lumen pH cannot properly increase; or in Zollinger–Edison syndrome, where the increased acid secretion from the stomach has a similar consequence.
3. Obstruction of the pancreatic duct by pancreatic lithiasis due to chronic pancreatitis, pancreatic tumors, or ampulomas.
4. Decreased lipase stimulation and production, which has been described in patients with celiac disease, Crohn’s disease, or Shwachman–Diamond syndrome (rare autosomal recessive disorder, which is the second most common cause of exocrine pancreatic insufficiency after cystic fibrosis and caused by an SBDS gene mutation on chromosome 7 that induces an abnormal functioning of ribosomes. The usual presentation is exocrine pancreatic dysfunction, skeletal abnormalities, and bone marrow dysfunction). Diabetes mellitus affects pancreatic microvascularization after years. This might facilitate fibrosis of the gland and a loss of functional tissue.
5. Motility alterations, whereby partial or total gastrectomy eliminates the reservoir and the progressive arrival of chyme to the intestinal lumen, causing a decrease in stimulation through hormones and a reduction in pancreatic enzyme secretion. The prevalence of EPI after gastrectomy is as high as 40–80% [2].

Pancreas-related malnutrition is mainly due to the difficulty in digesting dietary fats caused by pancreatic lipase [102]. The pancreatic secretion of amylase and proteases can be reduced, but the digestion of carbohydrates and proteins is maintained thanks to the action of salivary, gastric, and intestinal enzymes [102]. On the other hand, pancreatic lipase is responsible for 40% to 70% of the hydrolysis of triglycerides, which are degraded to fatty acids and monoglycerides capable of forming micelles to be absorbed into the membrane of enterocytes at the level of the jejunum. Other enzymes cannot compensate for the loss of pancreatic lipase.

It is well established that EPI leads to malnutrition [103]. Likewise, it has been suggested that malnutrition can also cause EPI due to studies carried out in severely malnourished children, although the causal mechanism is not fully understood [104].

The theory tends to establish that EPI occurs when the damage to the pancreatic parenchyma is greater than 90%. However, it is likely that it occurs gradually, and there are several factors that may influence its development [105]. The prevalence of EPI in patients with CP is high (between 60% and 90% after 12 years from diagnosis) [2]. The pancreatic gland undergoes progressive inflammatory damage, which causes the progressive destruction of glandular tissue. Chronic inflammation induces pancreatic fibrosis, which keeps the tissue dysfunctional, thus affecting both exocrine and endocrine function [102]. The incidence of chronic pancreatitis in studies performed in Europe is about 7.8 cases/100,000 people [106], and this incidence seems to be increasing over the years [107] due to the increased physician suspicion and the availability of more diagnostic methods for chronic pancreatitis.

## 7. Consequences of Exocrine Pancreatic Insufficiency for Nutritional Status

Nutrition is a complex process that involves various systems and organs. The objective is the conversion of the energy found in food to mobilize our body and carry out all the functions that a human being is currently capable of. Energy is obtained through macronutrients (carbohydrates, lipids, and proteins), but with the essential help of micronutrients (essential elements, which are necessary in lower quantities, including vitamins and minerals, such as zinc, iron, or iodine).

Nutritional status is used to determine the degree of health from the nutritional point of view of the patient. There is no single tool for diagnosis. Rather, it depends on the medical history, diet, anthropometric examination, biochemical analysis, and functional tests. Some of the scores that have been recommended at the community level are the Malnutrition Universal Screening Tool (MUST), and the first part of the Mini-Nutritional Assessment (MNA) in the elderly population [108]. Likewise, the Subjective Global Assessment (SGA) is a useful tool. These scores usually encompass the BMI, the recent unplanned weight loss, the mobility, and the nutritional intake from the subjective point of view and can be integrated with anthropometric and/or biochemical measurements (arm circumference, albumin, prealbumin, etc.).

The first clinical manifestations of patients with EPI are due to fat malabsorption: steatorrhea, weight loss, abdominal pain, or distention. A high clinical suspicion is essential, because the symptoms can be similar to other gastrointestinal disorders. Sometimes, patients adapt their dietary habits to avoid or minimize symptoms, such as abdominal pain or diarrhea [103].

Due to the difficulty in the absorption of lipid nutrients, the serum levels of fat-soluble vitamins (vitamins A, D, E, and K) may decrease [3,102]. Vitamin D deficiency is the most common, being observed in more than half of the patients [109]. This may justify the high prevalence of osteopathy in patients with CP, with the presence of osteopenia or osteoporosis in up to 68.9% of these patients. Osteopenia and osteopathy are more frequent among smokers, compared to non-smokers (63% vs. 22.2%) [109]. The European guidelines recommend the use of bone densitometry (DXA) every two years to screen patients who are at increased risk of fractures [110]. Frequently, bone fractures are caused by low trauma, showing the underlying osteopenia. Vitamin A and vitamin E deficiencies are less frequent, affecting 35% and 18% of patients, respectively [109]. The water-soluble vitamins are not usually decreased in the serum, except folic acid in patients affected by a high alcohol consumption [3]. Total proteins, albumin, transferrin, retinol binding protein, and magnesium can be used as biochemical markers, because they are frequently altered in CP patients with EPI in comparison with patients without EPI [111].

Moreover, a weight loss may be a common reason for specialist consultation, and EPI should be included in the differential diagnosis, especially in patients with risk factors, like a high alcohol consumption or cigarette smoking. The prevalence of underweight patients with CP is between 8–39% [3]. Likewise, sarcopenia can be measured by CT scan, and its prevalence can reach up to 17% among CP patients [3]. Recently, sarcopenia has been associated with increased hospitalizations and a reduced survival rate in a prospective cohort study [4].

## 8. Assessment of Exocrine Pancreatic Function

The pancreatic exocrine function can be measured invasively and directly or non-invasively and indirectly. The advantage of indirect methods is the price and the ease of reproduction.

### 8.1. Direct Tests

Secretin-stimulated pancreatic juice measurement used to be performed using a double-lumen tube that was developed by Dreiling and bears his name. However, it has recently been shown that it can be performed by endoscopy. In the first case, a light is located in the antrum to suck gastric secretion, and another lumen is located in the duodenum at the level of the Treitz ligament to aspirate the pancreatic juice after an infusion of 0.2 µg of synthetic secretin (cholecystokinin can also be used). The contents of the duodenal lumen are aspirated at 0, 15, 30, 45, and 60 min after the secretin administration [2]. A bicarbonate concentration of less than 80 mEq/L is indicative of moderate EPI. A severe EPI is defined as a bicarbonate concentration of less than 50 mEq/L. Endoscopic measurement is performed in a similar way, which provides greater comfort for the patient, with similar results according to different studies [112].

### 8.2. Indirect Tests

The indirect tests used in the assessment of exocrine pancreatic function are:

1. Coefficient fat absorption (CFA), which is based on the classic Van Kamer test. It is considered the gold-standard, although it is an expensive and time-consuming method for measuring EPI. As a preliminary step, a specific diet of 100 g of fat must be administered for five days, collecting the feces of the last three days for analysis [102]. The result is the percentage of dietary fat that is absorbed, which is usually above 93% in non-EPI patients. The definition of steatorrhea is established when there is 7 g of fat in 24 h [3]. It is the only test approved by the American Food and Drug Administration (FDA) and the European Medicines Agency to assess EPI in clinical trials. However, it is not a widely used test in clinical practice. The most common criticism is that there is wide variability inside the test [3].
2. Fecal elastase, which is based on the measurement of a very stable enzyme (elastase-1) that is produced in the acinar cells of the pancreas, binds bile salts in the intestine with little degradation, and can be measured in the feces. It can be measured in a single stool sample, with the requirement that these must be solid stools, because liquid stools can underestimate the presence of the enzyme and have a false positive result. A concentration of less than 200 µg/g is considered pathological. The specificity of the method is 93%, with a sensitivity for moderate and severe PID close to 100% [2]. The sensitivity decreases to 63% in cases of mild EPI. There are several measurement methods: ELISA, through the use of monoclonal antibodies or the use of polyclonal antisera. Monoclonal measurements do not interfere with enzyme replacement therapy, while polyclonal ones do [2]. Given its good results and its comfort, it has become the most widely used diagnostic test for evaluating EPI.
3. Secretin-stimulated magnetic resonance cholangiopancreatography. Pancreatic magnetic resonance imaging has been shown to be useful for evaluating the pancreatic parenchyma, surrounding tissues, and, especially, the main pancreatic duct [113]. This allows for anatomical as well as functional assessment. Secretin stimulation enhances the enzyme secretion and visualization of the pancreatic duct. In this way, systems and scores have been developed to evaluate pancreatic function according to the increase in pancreatic juice, which can be measured by duodenal filling [2,112]. However, this diagnostic test should be standardized in a better way [110,112,114]. This method might also be useful in the assessment of the etiology of chronic pancreatitis [115].
4. Breath test with marked-13Carbon, which is carried out by administering triglycerides marked with 13C. Pancreatic lipase is capable of hydrolyzing triglycerides into fatty acids, thus releasing 13C. This can be detected by the breath test, which varies depending on the activity of the pancreatic lipase. Its disadvantage is its low specificity and sensitivity, especially in mild EPI cases. The advantage is that it is a dynamic test, because the exhalation curve can be drawn.

**Figure 2:**
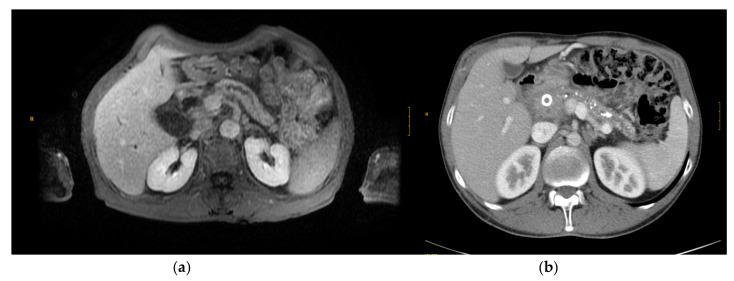
Radiological features of chronic pancreatitis. (**a**) MR image shows irregular pancreatic duct (PD) contour and ductal dilatation. (**b**) CT image also shows moderately to markedly irregular PD contour and ductal dilatation. Calcifications in neck and body of pancreas and a metallic biliary stent in head can be observed.

The currently recommended diagnostic method is the measurement of fecal elastase [110]. However, with suspected mild pancreatic insufficiency, direct methods, such as secretin-stimulated pancreatic juice measurement, may be helpful. Magnetic resonance imaging will be useful in the near future [110], but the availability in tertiary centers should increase (Figure 2).

## 9. Modern Management of Exocrine Pancreatic Insufficiency

The management of EPI is carried out through the oral Pancreatic Enzyme Replacement Therapy (PERT). Commercialized pancreatic enzyme tablets contain different doses of lipases, amylases, and proteases. The dosage of pancreatic lipase is the same as that of the reference. The enzymes, usually of animal origin, are included in enteric-coated microspheres. The capsule in which the microspheres are included is degraded by the acid upon reaching the gastric body, distributing homogeneously with the chyme and releasing the content after reaching the small intestine, when the pH rises above 5.5. There are different approved enzyme preparations, for which no comparative clinical trials have been developed [3,110].

Likewise, lifestyle changes are important for these patients, and smoking cessation, alcohol consumption reduction, and fat-soluble vitamin supplementation should be recommended, if necessary [102]. A low-fat diet is not recommended for these patients to avoid aggravating malnutrition [102,105], because approximately 30–33% of the energy is taken from fat in a normal diet [103]. In addition, a high fiber diet is not recommended, because fiber reduces lipase activity [103].

The initiation of PERT is indicated when diarrhea, steatorrhea, weight loss, or analytical signs of malnutrition are present [110]. The goal of treatment should be based on the improvement of malabsorption symptoms and nutritional deficits. The efficacy of PERT with the treatment of EPI has been demonstrated through meta-analyses of clinical trials, reducing fecal fat and nitrogen excretion and abdominal pain, compared to a placebo [116]. The fecal consistency tended to improve, but no improvement in stool frequency or flatulence was observed [116]. Another PERT advantage is the safety that it has shown, without showing a difference in side effects, compared to a placebo, in almost every study carried out [117].

The classic initial dose is 500 lipase units/kg body weight in each main meal [3], which has been standardized to 40,000–50,000 Ph. U in the recommendation of the European CP guidelines [110]. The dosage is halved for side meals or snacks. It has been suggested that the effectiveness may be higher if the dose is administered directly after meals, rather than before meals [116]. Physiologically, pancreatic lipase secretion is 9000–18,000 units per minute for about 4 h. Theoretically, it might be reasonable to guide pancreatic lipase toward 100,000 units per meal in cases of severe EPI. However, it has not been possible to demonstrate any significant improvement using different dosages of pancreatic enzymes in clinical trials [116].

The response to treatment is assessed clinically and analytically. Elastase is not usually useful in assessing the response to treatment, as we have seen previously. In cases in which it is necessary to assess the absence of a response, the fat absorption coefficient test or the marked carbon breath test could be used [110].

The absence of a response is treated with an increase in the dose of pancreatic enzyme, and it should be progressive. In addition, it is important to highlight that an excessively acid environment destroys pancreatic enzymes, despite the enteric coating. Thus, concomitant treatment with proton pump inhibitors can prevent this enzymatic destruction [118,119].

About 10–15% of patients with EPI will take enteral supplements because of malnutrition, and 5% will even require tube feeding (often nasojejunal) when oral nutrition fails due to abdominal pain, nausea, or vomiting [3]. A weight gain and improvement in pain have been achieved with tube feeding in patients with CP [3]. RELiZORB (Alcresta Therapeutics, Inc.; Newton, MA, USA) is a new device that may be helpful when cystic fibrosis patients require an enteral tube. It is a single-use cartridge with pancreatic lipase beads inside. Enteral nutrition is introduced through this cartridge, thus hydrolyzing triglycerides to absorbable forms, before the formula reaches the intestinal lumen.

In conclusion, nutrition in patients with pancreatic pathologies is essential for the improvement of their symptoms, their quality of life, and the correction of their nutritional deficits. The nutritional status should be systematically assessed in such patients during follow-up due to the pancreatic disease. Oral Pancreatic Enzyme Replacement Therapy is a cornerstone in the management of these patients. PERT should be prescribed in the correct doses and adjusted according to the clinical results obtained. We must not forget that changes in lifestyle, a correction of nutritional vitamin deficiencies, and the prevention of fractures due to osteopenia are all part of the treatment. Diagnostic imaging methods will probably improve in the future, and they could assess both the anatomy and the functionality of the pancreatic gland.

## 10. Conclusions

In conclusion, nutrition in patients with liver and pancreatic diseases is essential for the improvement of clinical outcomes, symptoms, quality of life, and the correction of nutritional deficits. While the spectrum of these diseases is wide, and the mechanisms of the onset of malnutrition are numerous and interrelated, clinical and nutritional manifestations are common. Despite the fact that recent progress has been made and the pathophysiological mechanisms have been better established, the impact on nutritional status remains a challenging problem, with little guiding knowledge. The nutritional status of all these patients should be systematically assessed. Even though multiple tools are available, the screening and diagnosis of malnutrition in patients with liver and pancreatic diseases are complex. In both settings, a change in lifestyle and correction of nutritional vitamin deficiencies are crucial parts of the treatment. The main nutritional strategies to overcome the nutritional consequences of liver and pancreas disease include nutritional counselling, frequent feeding, and nutritional supplementation. Finally, PERT has dramatically changed the management and outcomes in patients with EPI.

## Figures and Tables

**Table 1:** Consequences of liver disease on nutritional status.

Nutritional Consequence [Ref.]	Mechanisms in Chronic Liver Disease
1. Impaired dietary intake [19,20]	Anorexia, dysgeusia, abdominal pain, bloating, early satiety secondary to ascites, prescription of restrictive diets, alcohol consumption
2. Altered macro and micronutrient metabolism [13,14,15,16,21,22,23,24]	Lack of glycogen and vitamin storage, breakdown of fat and proteins as the principal energy source, decrease of vitamin and mineral levels
3. Energy metabolism disturbances [25]	Hypermetabolic state, impaired glucose and lipid metabolism, sedentary lifestyle
4. Increase in energy expenditure [26,27]	Increased catecholamines, malnutrition, immune compromise
5. Nutrient malabsorption [28,29]	Decreased bile production, cholestasis, portosystemic shunting, portal hypertension gastropathy and enteropathy, small intestinal bacterial overgrowth, drug-related diarrhea
6. Sarcopenia and muscle function [30,31,32]	Proteolysis as the energy source, inhibition of muscle growth, muscle autophagy, proinflammatory state
7. Metabolic osteopathy [33]	Decrease in bone formation, increased bone resorption, dysbiosis, vitamin K and D deficiencies

**Table 2:** Role of vitamins and minerals in the liver (RBP4: Retinol Binding Protein 4. HSc: Hepatic Stellate cells. MAFLD: Metabolic Associated Fatty Liver Disease).

Vitamin [Ref.]		Liver Role	Deficiency and Liver Disease
Fat-soluble vitamins			
A (retinol) [14,15,16]		Production of RBP4 (transporter) Main storage in HSc (80%)	Lost in vitamin A storage through the transformation of HSc into myofibroblasts. Deficiency is associated with nyctalopia (night blindness) and with hepatic encephalopathy
D [14,15,16,22]		25-hydroxylation site Production of binding proteins	Deficiency is associated with fibrosis, liver dysfunction, and mortality
K [14,15,16]		Absorption of vitamin K trough bile acids	Deficiency is associated with coagulopathy and bone disease through an inadequate carboxylation of bone matrix proteins
E [14,15,16]		Absorption of vitamin E trough bile acids	Deficiency is associated with hemolytic anemia, creatinuria, and neuronal degeneration
**Water-soluble vitamins**			
B [14,15,16]	B1 (thiamine)	Normal thiamine function	Lost in activation and transport. Deficiency is associated with neurologic dysfunction (Wernicke encephalopathy) and high-output heart failure (wet beriberi)
	B2 (riboflavin)	Storage of riboflavin	Inadequate intake, increased utilization, and deficient storage. Deficiency is associated with inflammation of the gums and sores
	B6 (pyridoxine)	Storage of pyridoxine	Deficiency is associated with anemia and neutropenia
	B9 (folate)	Storage of folate	Deficiency is associated with anemia and macrocytosis
	B12 (cobalamin)	Storage of cobalamin	Deficiency is associated with anemia and neutropenia
C [14,15,16]		Storage of vitamin C	Deficiency is common in MAFLD. Deficiency is associated with bleeding, joint pain, and an increase of free radicals
**Minerals**			
Zinc (Zn) [14,15,16]		Absorption of Zn	Inadequate dietary intake, impaired absorption, and an increase in urinary loss. Deficiency is associated with hepatic encephalopathy and alterations in taste and smell
Magnesium (Mg) [14,15,16]		Transport of Mg	Impaired transport and decrease intake. Deficiency is associated with dysgeusia, decreased appetite, muscle cramps, and weakness
Manganese (Mn) [23]		Absorption trough bile acid production	Elevated if there is a decrease in biliary excretion Deficiency is associated with brain accumulation and parkinsonism
Carnitine [24]		Metabolism of carnitine	Poor intake. Deficiency is associated with muscle cramps
Selenium (Se) [14,15,16]		Metabolism of Se	Deficiency related to severity liver disease Deficiency is associated with insulin resistance
Iron (Fe) [14,15,16]		Metabolism of Fe	Overload in alcoholic liver disease. Deficiency is associated with hepatic overload, fibrosis, and dysfunction

**Table 3:** Most frequently used screening tools for patients with liver cirrhosis.

Screening Tool [Ref.]	Target Population	Variables	Strengths and Weaknesses	Usefulness in Patients with Liver Cirrhosis
MST [40]	Hospitalized patients	1—Weight loss 2—Food intake 3—Appetite	Quick and easy No calculations No training Self-administered	May be inaccurate due to fluid overload. Low sensitivity in patients with liver cirrhosis.
MUST [41]	Hospitalized patients and outpatients	1—BMI 2—Weight loss 3—Acute illness and impact on dietary	Quick and easy Adds acute illness Offers advice	May be inaccurate due to fluid overload. Low sensitivity in patients with liver cirrhosis.
MNA-SF [42]	Elderly patients	1—Weight loss 2—Appetite 3—Mobility 4—Neuropsycho problems 5—BMI 6—Acute illness	Full evaluation, not only nutritional aspects BMI can be replaced by calf diameter	Good performance in liver cirrhosis. High sensitivity and good specificity.
NRS-2002 [43]	Hospitalized patients	1—BMI 2—Weight loss 3—Food intake 4—Illness severity	Adds illness severity and age	May be inaccurate due to fluid overload. Low sensitivity in liver cirrhosis. High specificity
CONUT [44]	Informatic tool Hospitalized patients and outpatients	1—Albumin 2—Cholesterol 3—Lymphocytes 4—Age 5—Illness severity 6—Length of illness 7—Treatment	Automated screening of large populations *Blood test required* *Low specificity*	Predictor of survival and complications after liver resection. Predictor of survival in end-stage liver disease.
SNAQ [45]	Hospitalized patients and outpatients	1—Weight loss 2—Appetite 3—Nutritional supplements 4—BMI 5—Albumin 6—Lymphocytes	Simple and quick Provides a recommendation *Blood test required*	Limited data on the population with liver cirrhosis, but correlation with the Child–Pugh stage.
RFH-NPT [46]	Patients with liver cirrhosis	1—Transplant 2—Fluid overload 3—Weight loss 4—Food intake 5—BMI (in absence of fluid overload) 6—Acute illness	Adds transplantation Reduces the impact of fluid retention Adds acute illness	Superior results compared to other tests in liver cirrhosis. High sensitivity and specificity.
LDUST [45]	Patients with liver cirrhosis	1—Food intake, 2—Weight loss 3—Body fat loss 4—Muscle mass loss 5—Fluid overload 6—Functional capability	Reduces the impact of fluid retention Adds functional capacity *Includes subjective variables*	Limited data in clinical practice. High sensitivity and specificity.

Weaknesses appear in italics. Abbreviations: Malnutrition Screening Tool (MST); Malnutrition Universal Screening Tool (MUST); Nutrition Risk Screening (NRS-2002); Controlling Nutritional Status (CONUT); Short Nutritional Assessment Questionnaire (SNAQ); Royal Free Hospital-Nutrition Prioritizing Tool (RFH-NPT); Liver Disease Universal Screening Tool (LDUST).

**Table 4:** Normal pancreatic physiologic functions.

Normal Pancreatic Physiology Phases		Beginning	Secretion	Function	Neurotransmission and Hormones Involved
Digestive secretion	1. Cephalic phase	Before the food reaches the stomach	Acinar secretion	Pancreatic enzyme synthesis and moderate secretion	ACh (vagal nerve) VIP GRP
	2. Gastric phase	Gastric distension	Acinar secretion	Low pancreatic enzymes secretion with small amounts of water and bicarbonate.	Gastropancreatic vagovagal reflex
	3. Intestinal phase	When the chyme enters the intestinal lumen and pH < 4.5	Ductal secretion	Secretion of large amounts of fluid and bicarbonate and pancreatic enzymes.	Secretin (S intestinal cells) → bicarbonate Cholecystokinin (I intestinal cells) → enzymes
Interdigestive secretion		Between meals cyclically	Ductal secretion	Cleansing of excretion system	Ach, peptide motilin and pancreatic polypeptide

Abbreviations: acetylcholine (ACh); vasoactive intestinal peptide (VIP); gastrin-releasing peptide (GRP).
